# Graphene Bridge Heterostructure Devices for Negative Differential Transconductance Circuit Applications

**DOI:** 10.1007/s40820-022-01001-5

**Published:** 2022-12-29

**Authors:** Minjong Lee, Tae Wook Kim, Chang Yong Park, Kimoon Lee, Takashi Taniguchi, Kenji Watanabe, Min-gu Kim, Do Kyung Hwang, Young Tack Lee

**Affiliations:** 1 Department of Electrical and Computer Engineering, Inha University, Incheon, 22212 Republic of Korea; 2 Department of Materials Science and Engineering, The University of Texas at Dallas, Richardson, TX 75080 USA; 3 Center for Opto-Electronic Materials and Devices, Post-Silicon Semiconductor Institute, Korea Institute of Science and Technology (KIST), Seoul, 02792 Republic of Korea; 4 Department of Physics, Kunsan National University, Gunsan, 54150 Republic of Korea; 5 Advanced Materials Laboratory, National Institute for Materials Science, Tsukuba, 305-0044 Japan; 6 Department of Information and Communication Engineering, Inha University, Incheon, 22212 Republic of Korea; 7 Division of Nanoscience and Technology, KIST School, University of Science and Technology (UST), Seoul, 02792 Republic of Korea; 8 Department of Electronic Engineering, Inha University, Incheon, 22212 Republic of Korea

**Keywords:** Graphene bridge, Heterostructure device, Non-classical transfer characteristics, Multi-value logic inverter, Frequency tripler

## Abstract

Two-dimensional van der Waals (2D vdW) material-based heterostructure devices have been widely studied for high-end electronic applications owing to their heterojunction properties. In this study, we demonstrate graphene (Gr)-bridge heterostructure devices consisting of laterally series-connected ambipolar semiconductor/Gr-bridge/*n*-type molybdenum disulfide as a channel material for field-effect transistors (FET). Unlike conventional FET operation, our Gr-bridge devices exhibit non-classical transfer characteristics (humped transfer curve), thus possessing a negative differential transconductance. These phenomena are interpreted as the operating behavior in two series-connected FETs, and they result from the gate-tunable contact capacity of the Gr-bridge layer. Multi-value logic inverters and frequency tripler circuits are successfully demonstrated using ambipolar semiconductors with narrow- and wide-bandgap materials as more advanced circuit applications based on non-classical transfer characteristics. Thus, we believe that our innovative and straightforward device structure engineering will be a promising technique for future multi-functional circuit applications of 2D nanoelectronics.

**Supplementary Information:**

The online version contains supplementary material available at 10.1007/s40820-022-01001-5.

## Highlights

- Graphene (Gr)-bridge heterostructure, consisting of a laterally series-connected (cascade) ambipolar/Gr/*n*-type 2D van der Waals channel materials for ambipolar semiconductor-based high-end application devices was developed.
- Non-classical transfer characteristics (humped curve) in FET operation and negative differential transconductances were observed.
- Gr-bridge heterostructure device with PdSe_2_ (narrow bandgap) allows multi-value logic operation while WSe_2_ (wide bandgap) enables frequency tripler circuit operation.

**Supplementary Information:**

The online version contains supplementary material available at 10.1007/s40820-022-01001-5.

## Graphical abstract

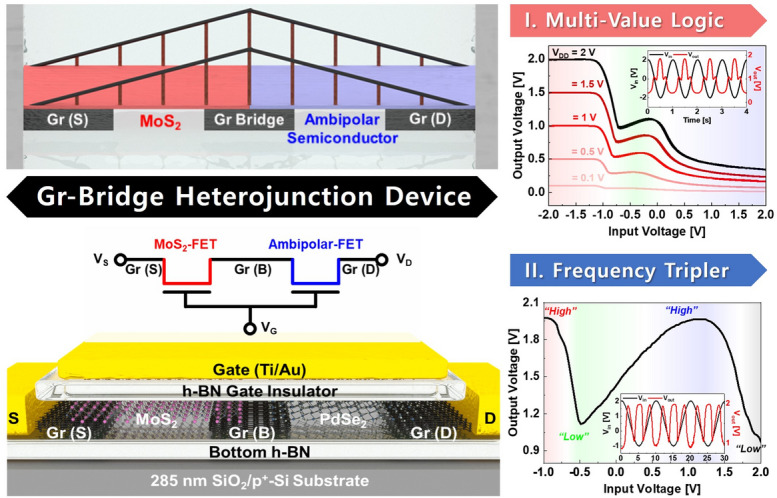

## Introduction

Two-dimensional van der Waals (2D vdW) nanomaterials have provided intriguing opportunities for various applications in nanoelectronics. For example, graphene (Gr) is a versatile material owing to its excellent carrier mobility and compatibility with various applications; however, the absence of a bandgap limits its use as a semiconductor channel beyond silicon-based electronics [1, 2]. Thus, it can be considered as a conductor material in future electronic devices, and several research groups have used exfoliated Gr as source (S), drain (D), and gate (G) electrodes to achieve all-2D material-based field-effect transistor (FET) applications [3–6]. The Gr S/D electrodes can effectively overcome the Schottky barrier, generally observed between 2D vdW semiconductors and metal contacts [7–10], owing to its gate-dependent Fermi level (*E*_F_) modulation [4, 11]. Because of this advantage, Gr electrodes are widely used in 2D vdW materials-based advanced electronic devices, thereby providing an innovative device structure and excellent device performance.

The 2D vdW semiconductors, such as transition metal dichalcogenides (TMDs) and 2D Xenes, have emerged because of their unique electrical properties since the discovery of Gr. Among these, tungsten diselenide (WSe_2_) and molybdenum ditelluride (MoTe_2_) possess strong gate-dependent characteristics; essentially, they exhibit ambipolar properties in FET applications [12, 13]. The tunable bandgap of WSe_2_ and MoTe_2_ is investigated as 1.22 (bulk) to 1.64 eV (monolayer) [14, 15] and 0.9 (bulk) to 1.1 eV (monolayer) [16, 17], respectively. In contrast, black phosphorus (BP) and palladium diselenide (PdSe_2_), which have narrower bandgaps, exhibit stronger ambipolar properties than WSe_2_ and MoTe_2_ active channels; therefore, they allow the realization of high-performance device applications. The tunable bandgap of BP and PdSe_2_ is reported as 0.3 (bulk) to 1.0 eV (monolayer) [18–20] and near-zero (bulk) to 1.3 eV (monolayer) [21–25], respectively.

These 2D vdW ambipolar semiconductors are expected to open new horizons for future nanoelectronics by developing new functionalized device applications, such as frequency doublers [26–30], reconfigurable homojunction diodes [31–34], and security circuits [35]. In advanced studies on 2D vdW semiconductors, heterostructure devices constructed using various 2D vdW materials as lego-like building blocks have been explored by numerous research groups to fabricate creative device architectures and investigate their unique junction properties [36–39]. A representative assembled device is a vertically stacked heterojunction diode consisting of two 2D vdW semiconductors for photodiodes [40–43], light-emitting diodes [44, 45], Esaki diodes [46–48], and extraordinary applications [49, 50].

In this study, a laterally series-connected ambipolar semiconductor/Gr/*n*-type molybdenum disulfide (MoS_2_) cascade channel device, called a Gr-bridge heterostructure, was studied beyond heterostructure junction device applications. Each active channel part exhibits distinct transport characteristics, such as ambipolar (PdSe_2_ or WSe_2_), mostly metallic (Gr-bridge), and unipolar (MoS_2_) properties. However, the series-connected cascade channel combines the transport characteristics of each part, thereby obeying the largest resistance among the channel materials, i.e., the total resistance of the Gr-bridged cascade channel. The Gr-bridge reduces the potential barrier height between the ambipolar semiconductor and *n*-type MoS_2_ junction region owing to its metallic and gapless energy band properties. Using this approach, the Gr-bridge allows the realization of unique switching devices and advanced application methods. We successfully demonstrated a multi-value logic inverter circuit and a frequency tripler for advanced electronic applications. Thus, we believe that the Gr-bridge heterojunction structure will open the gate for future electronics by designing device architectures and blending electrical properties toward high-end applications.

## Experimental Section

### Device Fabrication

The 285 nm SiO_2_/*p*^+^-Si substrate was ultrasonically cleaned in acetone, methyl alcohol, and isopropyl alcohol for 15 min each. To construct the Gr-bridge structure, polydimethylsiloxane (PDMS) stamps were used to exfoliate and transfer 2D vdW nanomaterials onto the substrate through the direct imprinting method. Next, a lift-off process was employed to form the top-gate electrode and the extended S/B/D pad electrodes. E-beam lithography and evaporation were, respectively, used for patterning and depositing metal electrodes at the 3D Convergence Center of Inha University.

### Electrical Characterization

All transfer, output, and VTC characteristics were measured using a semiconductor parameter analyzer (4156B, Agilent) in a dark probe station at room temperature (300 K). To demonstrate the dynamic performance of the WGM-FET, sinusoidal and ramp waveform signals were generated from a function generator (AFG31022, Tektronix).

## Results and Discussion

### 2D Ambipolar Semiconductor and Gr-Bridge Heterostructure Device

**Fig. 1:**
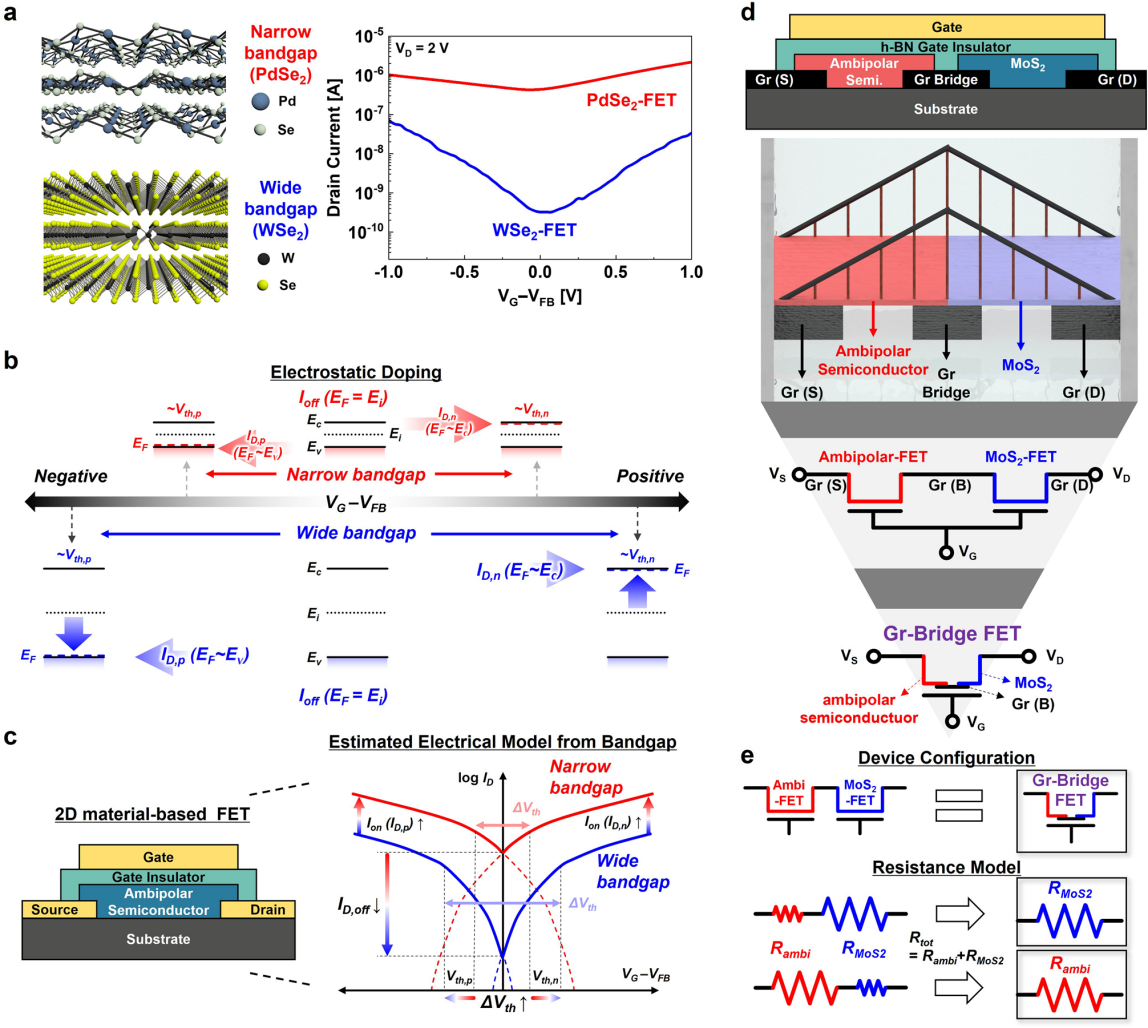
**a** Atomic crystal structures and *I*_D_–*V*_G_ transfer characteristic curves of the ambipolar PdSe_2_- and WSe_2_-FETs. **b** Schematic of the relationship between *E*_g_, *V*_th_, and *I*_D_ in ambipolar semiconductors. **c** Valley-like estimated electrical transfer model of 2D ambipolar semiconductor-based FETs according to the different energy bandgap properties (narrow and wide bandgap). **d** Conceptual device schematics and suggested electronic component symbol of the Gr-bridge heterostructure device (Gr-bridge FET). **e** Device configuration and resistance-in-series models of Gr-bridge FET

Figure 1a shows the transfer characteristics of PdSe_2_ (narrow bandgap, ~ 0.01 eV) and WSe_2_ (wide bandgap, ~ 1.3 eV) channel-based conventional FET devices [15, 24]. The hexagonal boron nitride (h-BN) sandwich structure and Gr S/D electrodes were adopted to investigate their fundamental ambipolar transfer characteristics (*I*_D_–*V*_G_) (see Fig. S1 for the cross-sectional device schematics and optical microscopy (OM) images of the two ambipolar-FET devices). The WSe_2_-FET exhibits both lower drain ON (*I*_D,*p*_ and *I*_D,*n*_) and OFF *I*_D_ (*I*_D,off_) current levels; however, the ON/OFF *I*_D_ ratio (*I*_ON_/*I*_OFF_) is higher than that of the PdSe_2_-FET. The narrow-bandgap materials typically have good electron and hole carrier mobilities; therefore, they bring a higher ON *I*_D_ value in FET devices but poor OFF *I*_D_ in general. These distinct transfer characteristics are due to their energy bandgap properties, as shown in Fig. 1b.

In 2D vdW ambipolar semiconductors, the gate voltage (*V*_G_) can electrostatically control the carrier concentration in the active channel of FET devices, namely the electrostatic doping effect [51]. A positive *V*_G_ over the threshold voltage of the *n*-type region (*V*_th,*n*_) causes *E*_F_ to reach the conduction-band edge (*E*_c_), whereas a negative *V*_G_ near the *V*_th_ of the *p*-type region (*V*_th,*p*_) enables *E*_F_ to reach the valence-band edge (*E*_v_) of ambipolar semiconductor channel materials [24, 52, 53]. Consequently, a wider bandgap will result in a larger valley-like transfer characteristic curve because it needs a stronger *V*_G_ (gate field) to modulate the *E*_F_ from the intrinsic Fermi level (*E*_i_) to *E*_c_ for the *n*-type transition (or the *E*_F_ from *E*_i_ to *E*_v_ for *p*-type transition) with a higher *I*_ON_/*I*_OFF_ ratio. *E*_G_ values of the ambipolar channel materials can be easily estimated by using the *V*_th_ difference (Δ*V*_th_ = *V*_th,*n*_-*V*_th,*p*_) and the average subthreshold slopes [SS_avg_ = (SS_*n*_ + SS_*p*_)/2] of the *n*-type and *p*-type regions by Eq. (1), where q is the electron charge and SS_60_ is the ideal SS value of 60 mV dec^−1^ [24, 35, 52, 53].

1 $$ E_{{\text{g}}} = \frac{{q\Delta V_{{{\text{th}}}} }}{{{\text{SS}}_{{{\text{avg}}}} {\text{/SS}}_{60} }} $$

Figure 1c shows the schematic of a typical top-gate transistor device and the expected valley-like transfer characteristic model of ambipolar FETs. The narrow bandgap of the active channel implies that even a small change in *V*_G_ leads to a large number of carriers in the active channel with *V*_th,*n*_ and *V*_th,*p*_ near the transition point (center of the valley-like transfer curve), thereby resulting in a high *I*_ON_, high *I*_OFF_, and low *I*_ON_/*I*_OFF_ ratio. Although a narrow-bandgap ambipolar semiconductor typically exhibits high-performance FET behavior, the inevitably high *I*_OFF_ renders it difficult to use for digital logic circuit applications with better switching characteristics, as compared to wide-bandgap ambipolar semiconductors [35]. Based on the ambipolar properties of PdSe_2_ (narrow bandgap) and WSe_2_ (wide bandgap) active channel materials, Gr-bridge heterostructure devices have been studied to achieve advanced electronic applications, such as multi-value logic inverters and frequency tripler circuits.

Such devices were formed as a platform of sequentially connected Gr-S, ambipolar semiconductors, Gr-bridge layers, MoS_2_, and Gr-D, as shown in Fig. 1d. The Gr bridge layer allows for the inherent characteristics of each 2D vdW semiconductor because it can provide tunable contact properties to both 2D vdW active channels according to its gate-dependent Fermi level modulation [4, 11]. Essentially, the Gr-bridge FET can be regarded as a series connection of the ambipolar-FET and MoS_2_-FET, with the same electrical characteristics. Figure 1e shows the simple resistance-in-series model of Gr-bridge FET. The total resistance (*R*_tot_) depends on the sum of the resistance in each FET part owing to the series connection properties; therefore, *R*_tot_ will follow the higher resistance of the two active channels during the device operation. Based on these operating properties, the connected ambipolar and MoS_2_ active channel devices in series will exhibit synthetic transfer characteristic curves as a breakthrough for high-end device applications.

### PdSe_2_-Gr-MoS_2_ Heterostructure FET

**Fig. 2:**
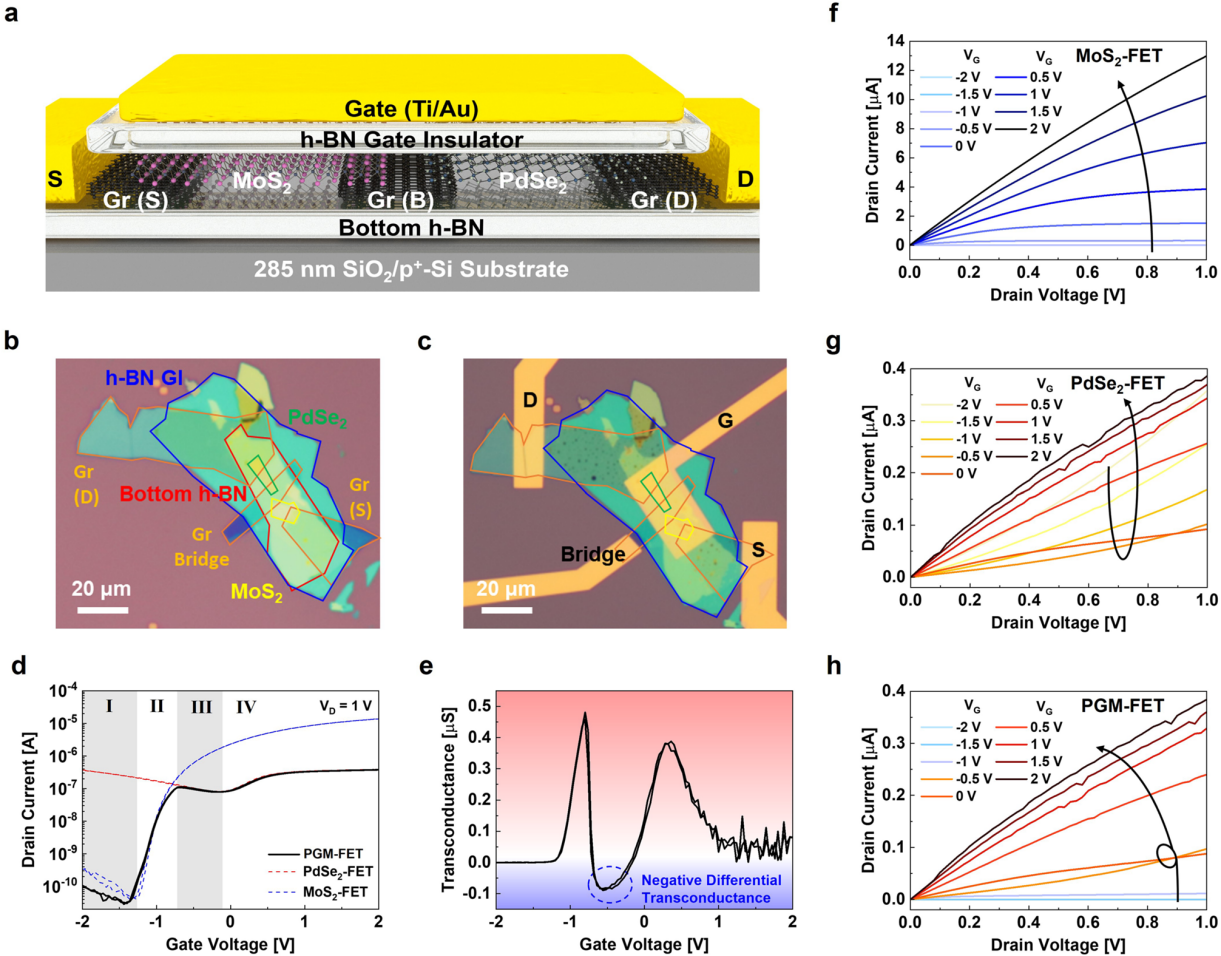
**a** Cross-sectional 3D device schematic of the PdSe_2_-Gr-MoS_2_ heterostructure FET (PGM-FET) for multi-value logic applications. OM images of the PGM-FET **b** before and **c** after the extended metal electrode fabrication. **d** *I*_D_–*V*_G_ transfer characteristic curves of the MoS_2_-FET (blue dashed line), PdSe_2_-FET (red dashed line), and PGM-FET (black solid line) at *V*_D_ of 1 V. **e** Estimated transconductance of the PGM-FET obtained from I_D_–V_D_ output characteristic curves of **f** MoS_2_-FET, **g** PdSe_2_-FET, and **h** PGM-FET. (Color figure online)

The first Gr-bridge-based high-end device consists of ambipolar PdSe_2_ (narrow bandgap) and *n*-type MoS_2_ active channel materials for multi-value logic applications, as shown in Fig. 2a. Figure 2b, c shows the OM images before and after metal patterning for the extended S/D and common gate (G) electrodes. For sample preparation, the bottom h-BN, Gr S/D, MoS_2_-Gr-PdSe_2_ heterostructure, and h-BN gate insulator were sequentially exfoliated and transferred onto a 285 nm-thick silicon dioxide (SiO_2_)/*p*^+^-silicon (Si) substrate. Subsequently, Ti/Au (5 /50 nm) electrodes were patterned and deposited using a combination of e-beam lithography and e-beam evaporation systems. The detailed step-by-step fabrication flow and thickness information for each 2D vdW material are depicted in Fig. S2 and S3, respectively.

Figure 2d shows the *I*_D_–*V*_G_ transfer curves of the MoS_2_-FET (blue dashed line), PdSe_2_-FET (red dashed line), and PdSe_2_-Gr-MoS_2_ heterostructure devices (solid black line) at a drain voltage (*V*_D_) of 1 V. We named the Gr-bridge device (PdSe_2_-Gr-MoS_2_ FET) “PGM-FET.” Because the h-BN sandwich structure was adopted to provide high-quality interfaces, hysteresis-less ideal transfer properties exist in all FET operations [54–57]. The MoS_2_-FET exhibits strong *n*-type transfer characteristics, and the PdSe_2_-FET shows ambipolar transfer characteristics using the Gr bridge layer as a source. Because the graphene interlayer has a gate-tunable contact capacity, it can act as a “bridge,” thereby reducing the Schottky junction properties between the ambipolar and *n*-type active channels. The detailed Raman spectrum analysis to confirm the clean and non-interactive interface properties of Gr-bridge and TMDC channels are shown in Fig. S4. Owing to the effect of the Gr bridge interconnection, the PGM-FET exhibits a tilde ( ~) symbol-like humped transfer characteristic curve: it traces the lower *I*_D_ of either the PdSe_2_ or MoS_2_ FETs according to the swept *V*_G_ (see the solid black line in Fig. 2d). The *I*_D_ of the PGM-FET follows the *I*_OFF_ (region I) and subthreshold *I*_D_ (region II) of the MoS_2_-FET, while regions III and IV, respectively, denote the case in the transfer *I*_D,*p*_ and *I*_D,*n*_ of PdSe_2_.

To analyze the detailed transport properties of the PGM-FET, the transconductance (*g*_m_ = d*I*_D_/d*V*_G_) was calculated, and a negative *g*_m_ was observed in region III (humped curve), as shown in Fig. 2e. Figure 2f–h shows the output characteristic (*I*_D_–*V*_D_) curves of the MoS_2_-FET, PdSe_2_-FET, and PGM-FET obtained from *V*_G_ ranging from − 2 to 2 V in steps of 0.5 V. The Gr bridge provides tunable ohmic contact properties; therefore, the output curves of the MoS_2_ and PdSe_2_ active channels exhibit typical *n*-type and ambipolar transport properties, respectively. Moreover, the PGM-FET exhibits the composite output characteristics of the MoS_2_-FET (*V*_G_ range of − 2 to − 1 V) and PdSe_2_-FET (*V*_G_ range of − 0.5 to 2 V).

### PGM-FET-Based Multi-value Logic Circuit Applications

**Fig. 3:**
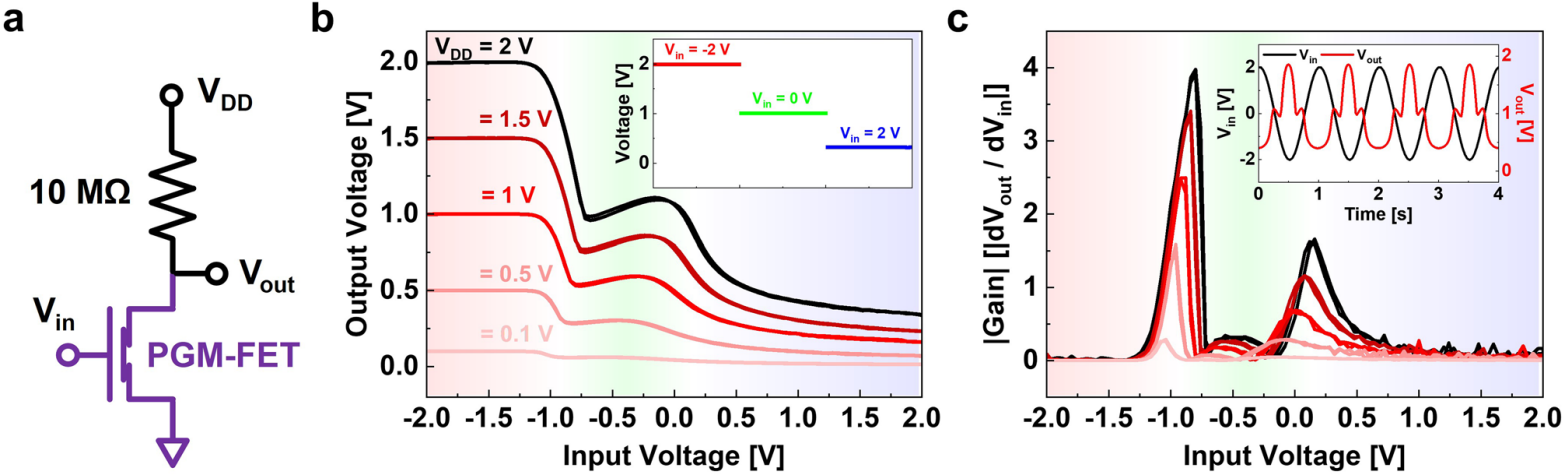
**a** Circuit diagram of PGM-FET-based resistive-load inverter for multi-value logic circuit applications. **b** VTC characteristic curves of PGM-FET-based multi-value logic operations for V_DD_ ranging from 0.1 to 2 V. The inset shows three distinguished output states (ternary logic states). **c** Voltage gain of the demonstrated ternary inverter logic circuit for V_DD_ ranging from 0.1 to 2 V. The inset shows the dynamic V_out_ responses (red) from the sinusoidal waveform V_in_ (black). (Color figure online)

Based on the non-classical humped *I*_D_–*V*_G_ transfer properties, we extended our PGM-FET study to multi-value logic circuit applications as a first-approach method. An external resistor of 10 MΩ was chosen for the resistive-load inverter circuit near the humped *I*_D_ curve, as shown in Fig. 3a. Figure 3b shows the voltage transfer characteristics (VTC) of the PGM-FET-based resistive-load inverter circuit under different supply voltage (*V*_DD_) conditions. The inset shows three distinct levels of ternary inverted output voltage (*V*_out_) responses. Input voltages (*V*_in_) of − 2, 0, and 2 V generate *V*_out_ of *V*_DD_, *V*_DD_/2, and approximately 0 V, respectively. Figure 3c shows the absolute voltage gain (|d*V*_out_/d*V*_in_|) of the ternary inverter logic circuit under different *V*_DD_ conditions. The voltage gain is approximately 4 (first voltage drop) and approximately 1.6 (second voltage drop) at a *V*_DD_ of 2 V. The inset shows the dynamic *V*_out_ responses obtained from a sinusoidal waveform of *V*_in_, which can identify the dynamic ternary levels of the demonstrated ternary logic circuit. The peak-to-peak voltage (*V*_*p*-*p*_) and periodic time (*T*) were 4 V and 1 s, respectively.

We developed inverted ternary logic for the Gr-bridge structure, which simply employs a narrow-bandgap ambipolar semiconductor (PdSe_2_) and an *n*-type semiconductor (MoS_2_). Based on the above understanding of the PGM-FET-based ternary logic circuit, we attempted to investigate a direct PdSe_2_-MoS_2_ junction FET (PMJ-FET) for a more advanced and practical device model, as shown in Fig. S5. Although the PMJ-FET also exhibits a non-classical humped *I*_D_–*V*_G_ transfer curve and ternary logic circuit operation, the absence of the Gr-bridge interlayer results in rectifying properties at the PdSe_2_/MoS_2_ junction [58]. Consequently, the PMJ-FET exhibits asymmetric (having direction) device characteristics and a limitation in dynamic operation, whereas the PGM-FET exhibits symmetric (bidirectional) logic circuit operation properties. Furthermore, reliability problems remain in the PMJ-FET because controlling the optimized junction properties between the two different semiconductor active channels is difficult. As shown in Fig. S4d, the PMJ-FET does not trace the transfer curves of the MoS_2_-FET and PdSe_2_-FET because of the rectifying junction properties of MoS_2_/PdSe_2_ [42]. Therefore, adopting the Gr-bridge device structure will provide reliability and convenience for further study of high-end multi-value logic applications.

### WSe_2_-Gr-MoS_2_ Heterostructure FET Device

**Fig. 4:**
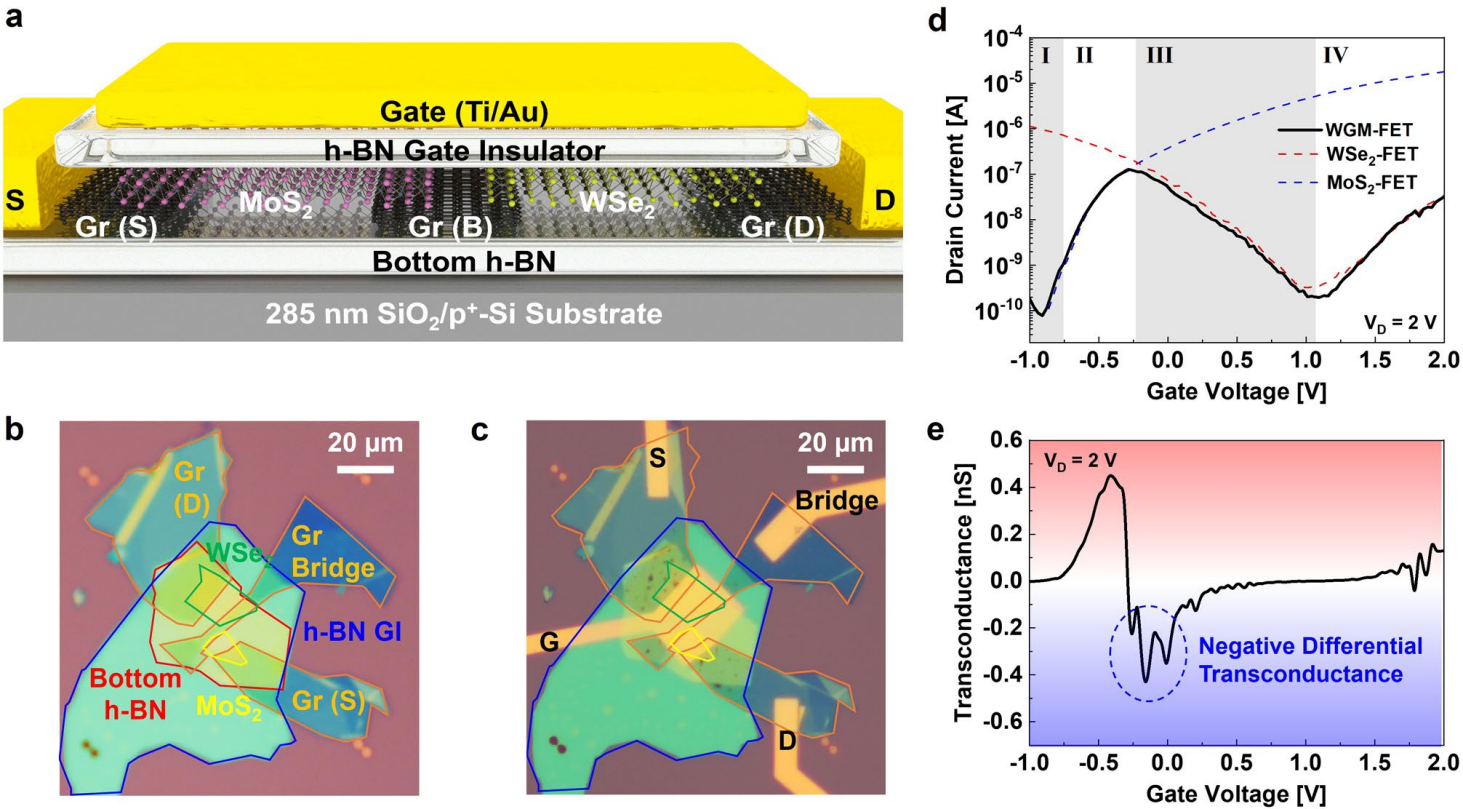
**a** Cross-sectional 3D device schematic of the WSe_2_-Gr-MoS_2_ heterostructure FET (WGM-FET) for frequency tripler circuit applications. OM images of the WGM-FET **b** before and **c** after the extended metal electrode fabrication. **d** *I*_D_–*V*_G_ transfer characteristic curves of the MoS_2_-FET (blue dashed line), WSe_2_-FET (red dashed line), and WGM-FET (black solid line). **e** Estimated transconductances of the WGM-FET obtained from **d**. (Color figure online)

As a second approach toward advanced electronic applications, WSe_2_, a wide-bandgap ambipolar active channel, was chosen to realize the Gr-bridge heterostructure device instead of the PdSe_2_-based ternary logic circuit. Figure 4a shows the 3D device schematic of WSe_2_-Gr-MoS_2_ FET, named “WGM-FET.” Figure 4b, c shows the OM images before and after patterning of the extended S/D and G electrodes, respectively, through the same device fabrication processes of PGM-FET. The detailed process flow and thickness information for each layer are shown in Figs. S6 and S7, respectively. Figure 4d shows the *I*_D_–*V*_G_ transfer curves of the MoS_2_-FET (blue dashed line), WSe_2_-FET (red dashed line), and WGM-FET (solid black line) at *V*_D_ of 2 V. In this case, the transfer curve of WGM-FET follows that of the MoS_2_-FET in regions I and II and that of WSe_2_-FET in regions III and IV. However, unlike the case of PGM-FET, the wider bandgap of the WSe_2_ channel allows a low *I*_OFF_ level of WGM-FET (wider region III); therefore, the transfer characteristic curves resemble an uppercase letter “N”. Figure 4e shows the *g*_m_–*V*_G_ curve of the WGM-FET; a negative *g*_m_ was observed in operation region III owing to the *p*-type transition properties of the WSe_2_ active channel.

### WGM-FET-Based Frequency Tripler Circuit Applications

**Fig. 5:**
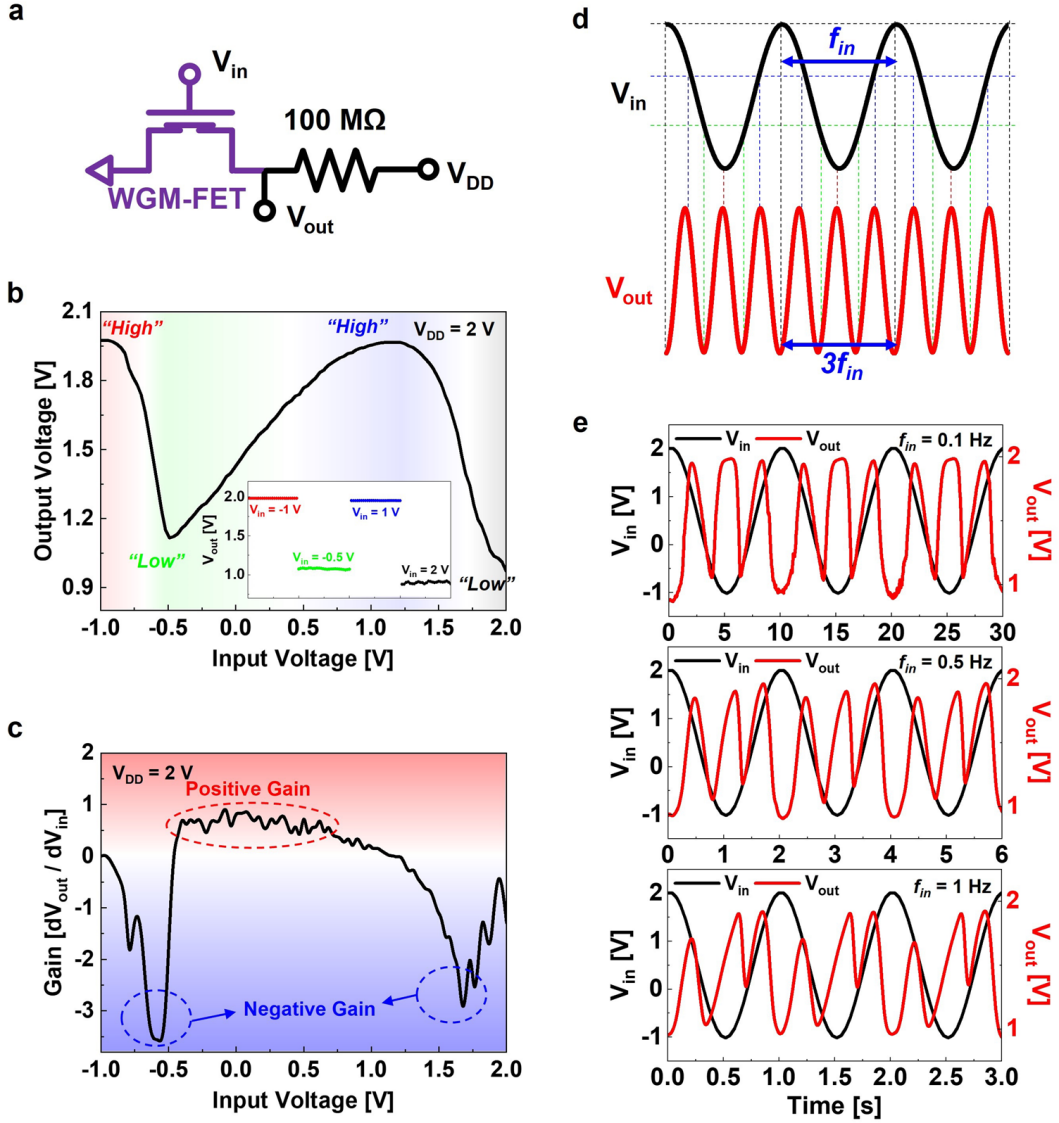
**a** Circuit diagram of the WGM-FET-based resistive-load inverter circuit applications. **b** Upside-down letter “N”-like VTC curve of WGM-FET-based frequency tripler circuit operations at *V*_DD_ of 2 V. The inset shows four distinguished output states, that is, sequentially repeated “High” and “Low” states. **c** Voltage gain of the demonstrated WGM-FET-based frequency tripler circuit. **d** Expected frequency response of frequency tripler (three cycles of *V*_out_) converted from a single cycle of *V*_in_. **e** Real-time *V*_out_ responses (red) of the frequency tripler circuit for 0.1, 0.5, and 1 Hz input sinusoidal waveform *V*_in_ (black). (Color figure online)

These non-classical transfer characteristics of the WGM-FET bring unique VTC characteristics of an upside-down letter “N”-like curve in inverter logic circuit applications, as shown in Fig. 5a. To achieve a resistive-load inverter circuit, an external resistor of 100 MΩ was connected to the WGM-FET. Figure 5b shows the VTC curve, and the four transition states were observed as sequentially “High”, “Low”, “High”, and “Low” output states according to the voltage sweep of *V*_in_ at *V*_DD_ of 2 V. The inset shows the four distinguished logic states at *V*_in_ of − 1, − 0.5, 1, and 2 V. Figure 5c shows the voltage gain of our demonstrated inverter circuit, which has both negative and positive values.

The *V*_in_ sweep from “Low (− 1 V)” to “High (2 V)” should produce sequential output states of “High–Low–High–Low” and the backward *V*_in_ sweep from “High” to “Low” generated the reversed output states of “Low–High–Low–High.” That is, a double sweep of *V*_in_ of “Low–High–Low,” similar to a single waveform, will produce the “High–Low–High–Low–High–Low–High” sequential output states. The repeatable “High” and “Low” output logic states enable an advanced frequency response application with respect to the sinusoidal waveform of *V*_in_, as shown in Fig. 5d. As a result, a single cycle of sinusoidal waveform *V*_in_ should produce three cycles of the waveform *V*_out_. This circuit application method can be regarded as a “frequency tripler,” which can generate the output frequency (*f*_out_) to triple the value from the input frequency (*f*_in_). Finally, for the first time, we successfully demonstrated a frequency tripler application with a single Gr-bridge heterostructure FET consisting of a wide-bandgap ambipolar semiconductor. Figure 5e shows the real-time *V*_out_ responses for the 0.1, 0.5, and 1 Hz sinusoidal waveforms of *V*_in_ (see Fig. S8 for the real-time *V*_out_ response for the 1 Hz ramp waveform *V*_in_). The *V*_out_ responses were analyzed using fast Fourier transform (FFT), as shown in Fig. S9. Our frequency response application should be economical to generate triple frequency toward low-power (frequency) and low-cost, more effective than the conventional frequency multiplier circuit for future advanced electronics.

## Conclusions

In this study, Gr-bridge FETs consisting of laterally series-connected ambipolar semiconductor/Gr-bridge/*n*-type MoS_2_ cascade channels were studied for high-end switching device applications based on their non-classical negative differential transconductance characteristics. The Gr-bridge layer could eliminate the Schottky junction properties between two semiconductor channels; therefore, the Gr-bridge FETs showed synthetic transfer characteristics, perfectly tracing the lower *I*_D_ of each channel material based on the simple resistance-in-series model, unlike the heterojunction devices without the Gr-bridge layer. Moreover, we successfully implemented two types of advanced electronic applications based on the bandgap properties of PdSe_2_ (narrow-bandgap) and WSe_2_ (wide-bandgap) ambipolar semiconductors for a multi-value logic inverter (PGM-FET) and frequency tripler (WGM-FET) circuits, respectively. Thus, we believe that the results of our Gr-bridge heterostructure devices and multi-functional circuit applications will provide reliability and convenience to open up a breakthrough toward future advanced electronics.

### Supplementary Information

Below is the link to the electronic supplementary material.

Supplementary file1 (PDF 962 KB)
