# Supplementary material for: Graphene Bridge Heterostructure Devices for Negative Differential Transconductance Circuit Applications
Source: Nanomicro Lett. 2022 Dec 29;15:22. doi: 10.1007/s40820-022-01001-5 (PMC9800667; doi:10.1007/s40820-022-01001-5)
Supplement: Supplementary file 1 — Supplementary file1 (PDF 962 KB) [file 40820_2022_1001_MOESM1_ESM.pdf]

Supplementary Information for

Graphene Bridge Heterostructure Devices for Negative Differential Transconductance Circuit Applications

Minjong Lee[1, 2, †], Tae Wook Kim[3, †], Chang Yong Park[1, †], Kimoon Lee[4], Takashi Taniguchi[5], Kenji Watanabe[5], Min-gu Kim[1, 6, *], Do Kyung Hwang[3, 7, *], Young Tack Lee[1, 8, *]

[1] Department of Electrical and Computer Engineering, Inha University, Incheon 22212, Republic of Korea

[2] Department of Materials Science and Engineering, The University of Texas at Dallas, Texas 75080, USA

[3] Center for Opto-Electronic Materials and Devices, Post-Silicon Semiconductor Institute, Korea Institute of Science and Technology (KIST), Seoul 02792, Republic of Korea

[4] Department of Physics, Kunsan National University, Gunsan, 54150, Republic of Korea

[5] Advanced Materials Laboratory, National Institute for Materials Science, Tsukuba 305-0044, Japan

[6] Department of Information and Communication Engineering, Inha University, Incheon 22212, Republic of Korea

[7] Division of Nanoscience and Technology, KIST School, University of Science and Technology (UST), Seoul 02792, Republic of Korea

[8] Department of Electronic Engineering, Inha University, Incheon 22212, Republic of Korea

[†] Minjong Lee, Tae Wook Kim and Chang Yong Park contributed equally to this work.

* Corresponding authors. E-mail: mgk@inha.ac.kr (Min-gu Kim), dkhwang@kist.re.kr (Do Kyung Hwang), ytlee@inha.ac.kr (Young Tack Lee)

Supplementary Figures

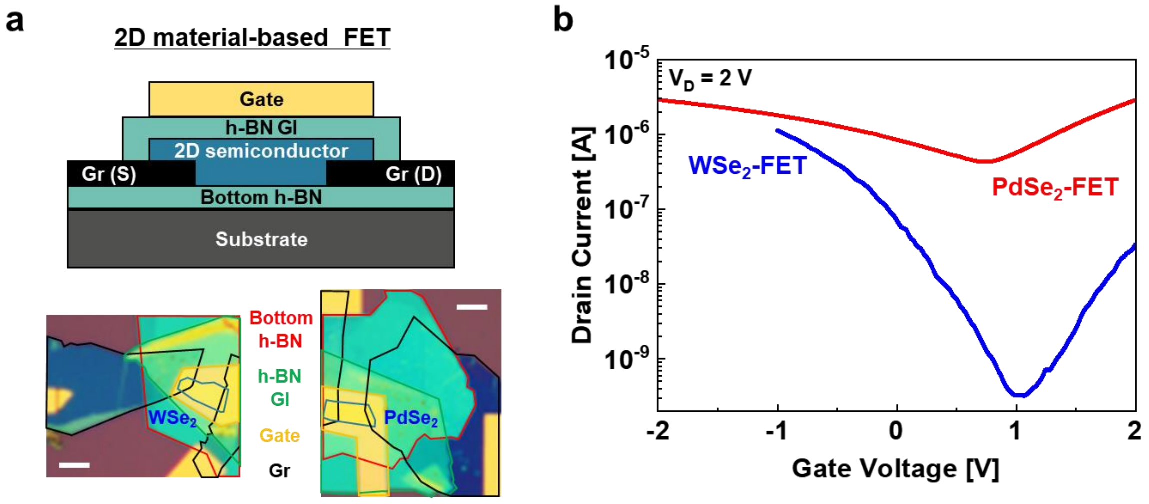

Fig. S1 WSe_2- and $PdSe_2$-based FET devices. **a** Cross-sectional schematic and OM images of the h-BN sandwiched WSe_2- and $PdSe_2$-FETs. **b** I_D–V_G transfer characteristic curves of the WSe_2-FET (blue line) and $PdSe_2$-FET (red line)

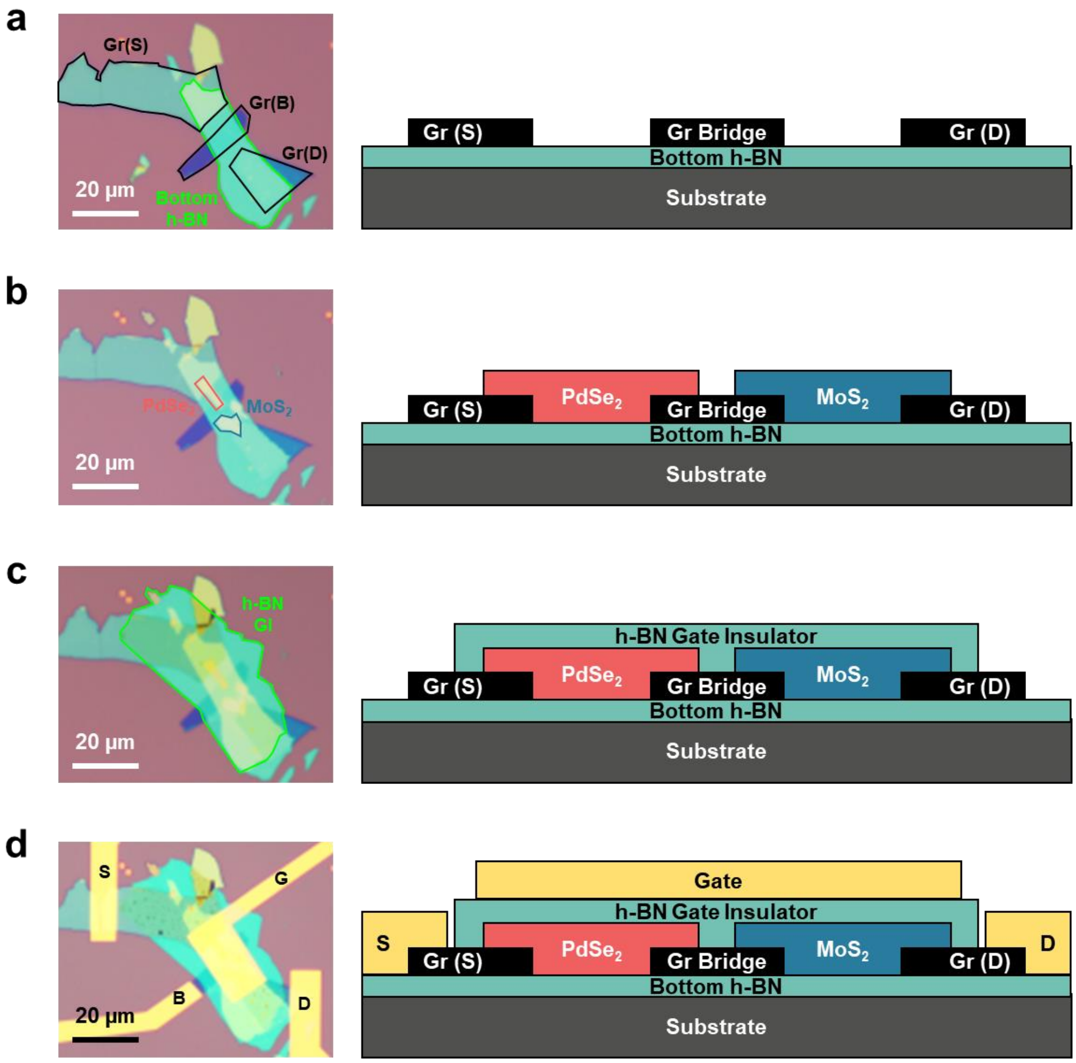

Fig. S2 Device fabrication flow of the PGM-FET. Device fabrication flow of the h-BN sandwiched PGM-FET as following: transferring **a** the bottom h-BN, Gr S/B/D, **b** $PdSe_2$, MoS_2, and **c** h-BN gate insulator; **d** patterning and depositing metal S/G/D electrodes

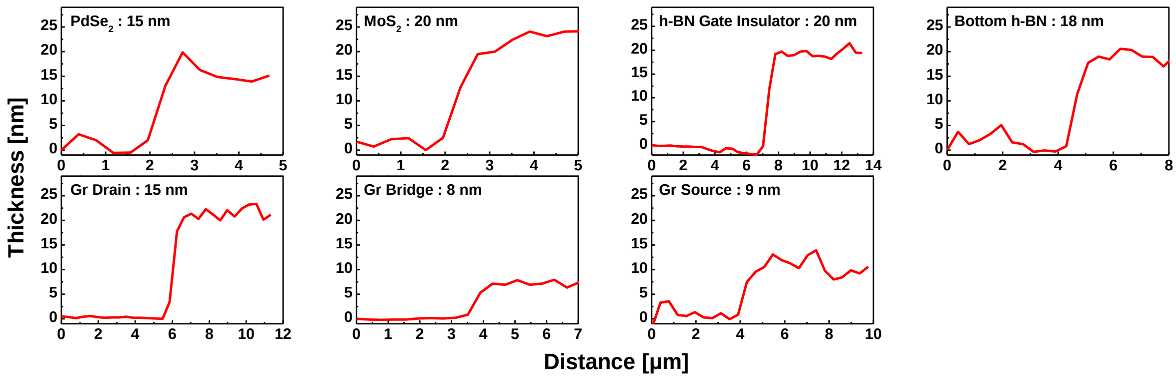

Fig. S3 Thickness of each flake in the PGM-FET. Step-height analysis of each flake in the PGM-FET by using atomic force microscopy (AFM)

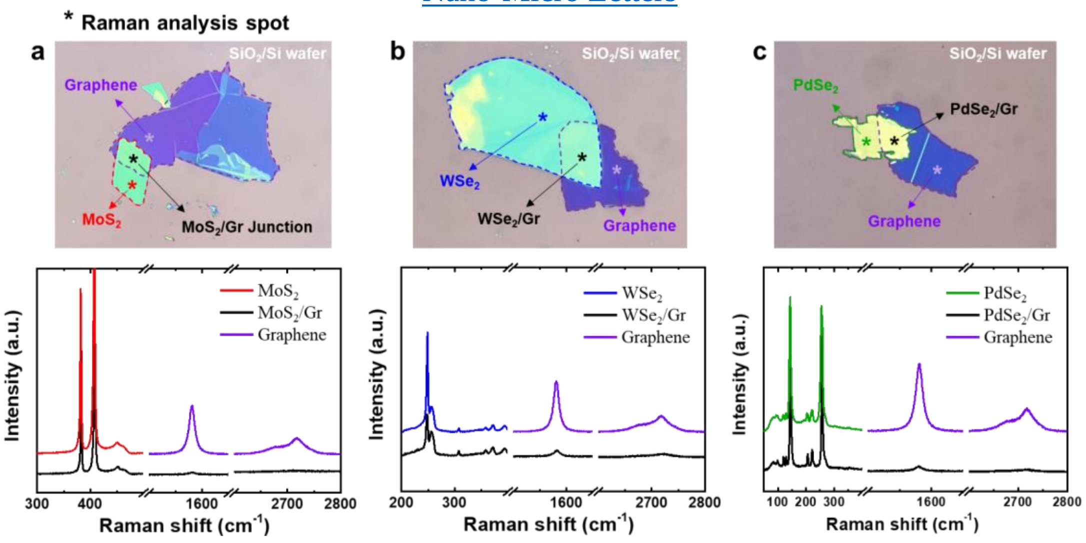

Fig. S4 Non-interative interface properties of Gr-bridge and TMDC channels. Raman spectrum analysis of **a** MoS_2/Gr, **b** WSe_2/Gr, and **c** $PdSe_2$/Gr junction devices on SiO_2/Si substrate

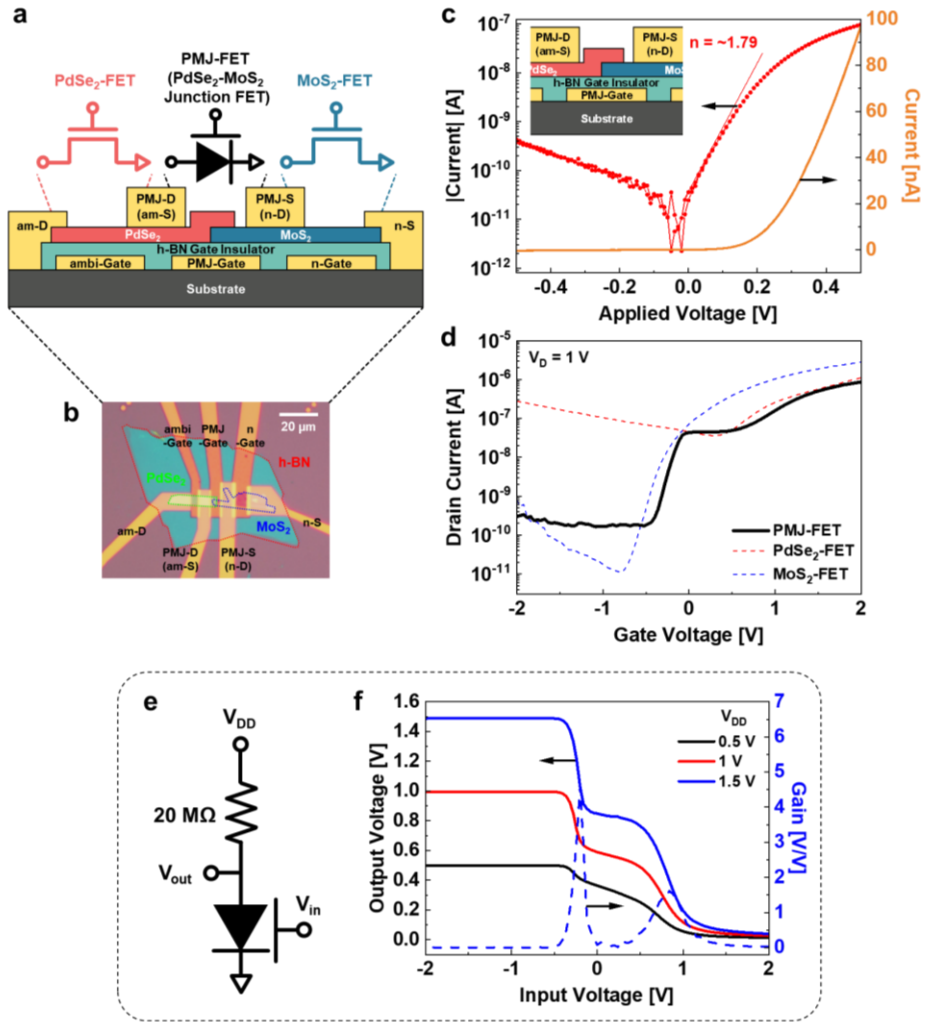

Fig. S5 Electrical characteristics of the $PdSe_2$-MoS_2 junction FET (PMJ-FET). a Cross-sectional schematic and the circuit symbol corresponding to each region in PMJ-FET. **b** OM image of the PMJ-FET. **c** I–V characteristic curve of the $PdSe_2$-MoS_2 heterojunction device, which shows the rectification properties. **d** I_D–V_G transfer characteristic curves of the MoS_2-FET (blue dashed line), $PdSe_2$-FET (red dashed line), and PMJ-FET (black solid line). **e** Circuit diagram of the PMJ-FET based inverter application with 20 MΩ load resistor. **f** VTC characteristic curves of the demonstrated circuit at V_{DD} of 0.5, 1, and 1.5 V

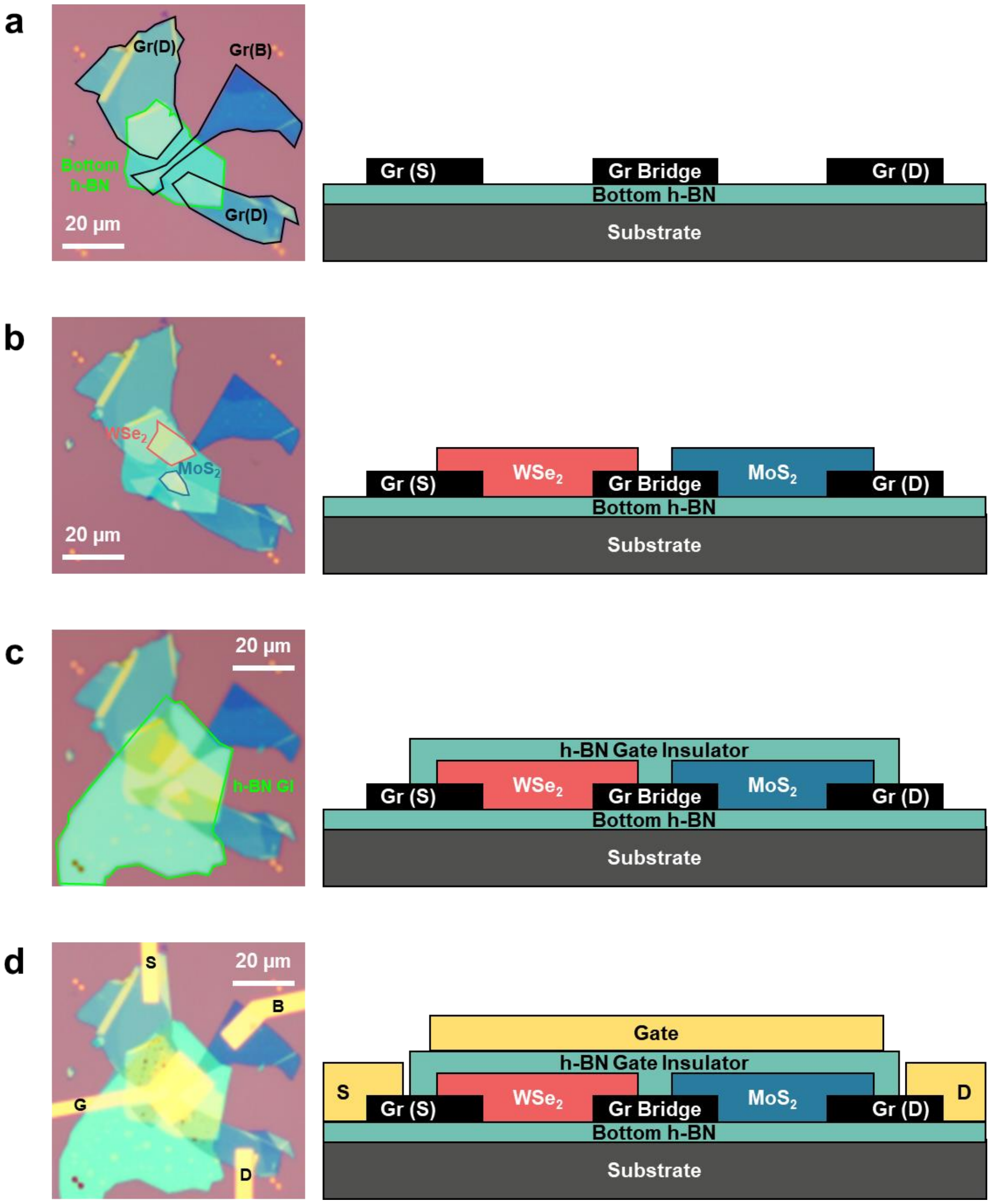

Fig. S6 Device fabrication flow of the WGM-FET. Device fabrication flow of the h-BN sandwiched WGM-FET as following: transferring **a** the bottom h-BN, Gr S/B/D, **b** WSe_2, MoS_2, and **c** h-BN gate insulator; **d** patterning and depositing metal S/G/D electrodes

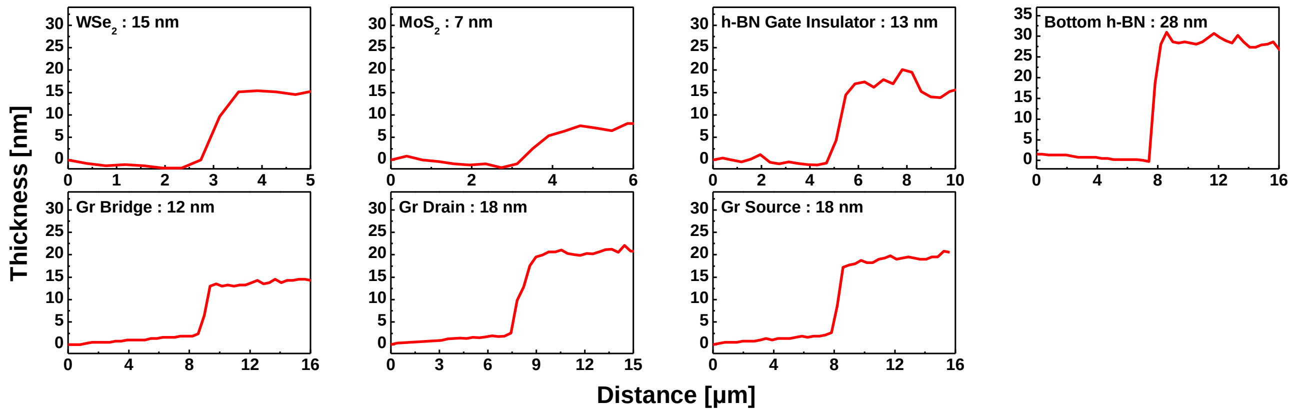

Fig. S7 Thickness of each flake in the WGM-FET. Step-height analysis of each flake in the WGM-FET by using AFM

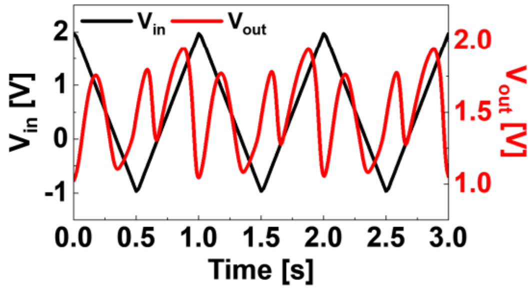

Fig. S8 Real-time V_{out} responses from ramp waveform V_{in}. Dynamic V_{out} responses from the 1Hz ramp waveform V_{in} ranging from –1 to 2 V

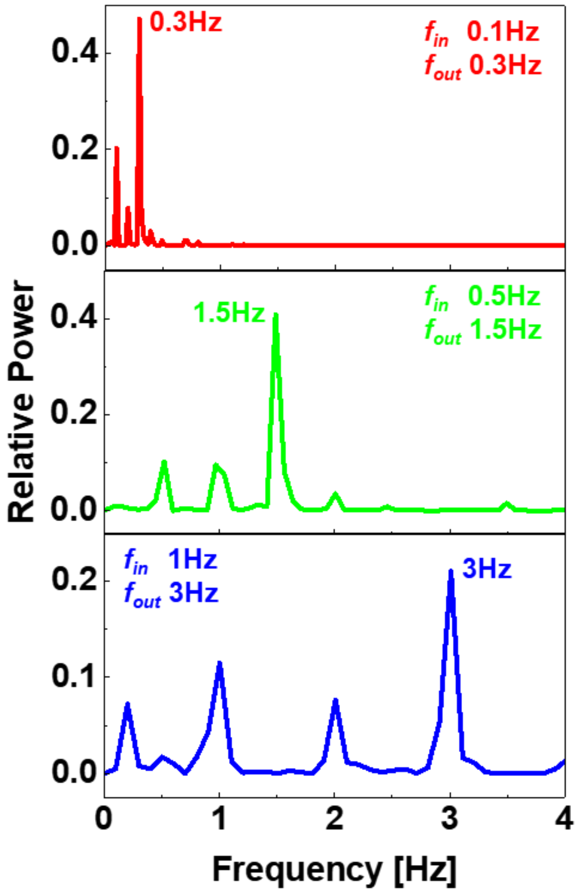

Fig. S9 Fast Fourier transform analysis of the frequency tripler application. Relative power spectrum of output signals converted by fast Fourier transform (FFT) method
